# Editorial: The effects of diet on health in insects

**DOI:** 10.3389/finsc.2023.1186027

**Published:** 2023-06-06

**Authors:** Christian W. W. Pirk, Ricarda Scheiner

**Affiliations:** ^1^ Social Insects Research Group, Department of Zoology & Entomology, University of Pretoria, Pretoria, South Africa; ^2^ Behavioral Physiology and Sociobiology, Theodor-Boveri-Institute, Biocenter, Julius-Maximilians-Universität Würzburg, Würzburg, Germany

**Keywords:** global change, pollination service, be a healthy insect, biodiversity, food security

Global change brings about a number of new challenges to insects. Urbanisation and landscape transformation, increasing temperatures, and frequent droughts will not only affect the species itself but also has knock-on effects throughout the trophic levels of a network. Plants might flower earlier and leave the previously default pollinator in a situation where it must look for a suitable alternative or starve. Long cold weather periods in spring after an early onset of flowering might starve both solitary and social insects in the beginning of the season. The increasing human food demand is resulting in agricultural intensification and an increase in monocultures, thereby reducing the diversity of floral nectar and pollen (1). Predatory and herbivore insects are similarly affected, with their favourite diet becoming desynchronised from their phenology, either temporally or spatially, due to changes in land use and the loss in connectivity linking favourable habitats.

Understanding how nutrition and insect health are linked is crucial to forecast the effects of global change on insect health and the consequences for biodiversity and food security, especially for beneficial insect species such as pollinators.

Generally, the dietary aspects of insects have mainly been approached from an integrated pest management or pest evaluation point of view. The investigation of nutrition in relation to insect health, especially for beneficial insects such as social bees, however, is still in its infancy. Although it is generally agreed that diverse landscapes are more profitable to social bees than monocultures, how the lack of pollen diversity affects colony development and reproduction is poorly understood.

Watrobska et al. investigate the important question of whether the protein content of pollen (monofloral pollen enriched with protein) or the diversity of pollen (polyfloral pollen) is more important for the development and reproduction of bumble bee colonies (*Bombus terrestris*). Their experiments clearly reveal that pollen protein content seems to be much more important for colony health and development than the diversity of pollen. However, the better development of their colonies fed with monofloral pollen might also be related to their choice of pollen, according to the authors. If their monofloral pollen had contained pollen from a different species, the results might have been different. After all, the pollen fed to bees needs to contain certain amino acids and fatty acids for a healthy diet. Overall, their experiments stress the great importance of high-protein content for a healthy diet in bumble bee colonies.

Frizzera et al. show a similar positive effect of a pollen-enriched diet on honeybees (*Apis mellifera*). Infestation with *Varroa destructor* generally leads to a faster behavioural maturation of the honeybee host (2), leading to a shorter life span (3). The increased behavioural maturation correlates with earlier changes in the expression of genes and hormones associated with task transition. The authors compare the gene expression of four genes, namely, *VG*, which encodes for vitellogenin, *jhe*, *apidaecin-1*, and *defensin-1*, and tested the abundance of DWV. When they fed additional pollen to Varroa-infested bees, they enhanced their lifespan by reversing the faster behavioural maturation induced by the parasite at the level of gene expression. The extended lifespan induced by the pollen-enriched diet further correlates with the positive effect of antimicrobial peptide gene expression and DWV (deformed wing virus) load, further reinforcing the beneficial effect of pollen. These data lay an important foundation for future analyses of the underlying evolutionary processes and applications to improve bee health.

The next article in this Research Topic investigates the importance of undernourishment during early larval stages in the honeybee (*Apis mellifera*). Schilcher et al. present evidence of how these social insects can buffer and potentially tolerate undernourishment during the larval stages. Bees raised under different dietary regimes show no differences in their behaviour, i.e., they initiate and finish nursing tasks and foraging at a similar age. The dietary regimes differed in the total amount that the larvae received. The undernourished group received 150 µl, the normal one received 160 µl, and the overfed group received 180 µl over a six-day period. This suggests that the effects of early larval undernourishment can be compensated for during early adulthood despite the measurable differences in body weight.

The ability to buffer periods of suboptimal nutrition is not restricted to social insects but can also be observed in the dietary plasticity of solitary insects. A fair number of studies investigating dietary effects have relied on artificial diets within a laboratory setup. In the case of lepidopteran models, the diet is specifically designed for ensuring optimal nutrition. It is, however, unclear to what degree the results obtained in the lab can be translated to field conditions under natural dietary conditions.

Costantin et al. compare the interaction of a plant-based diet and an artificial diet with a plastic immune strategy (density-dependent prophylaxis, DDP). They use the velvetbean caterpillar *Anticarsia gemmatalis*, which is known to adjust its immune defence depending on the conspecific density at which these caterpillars are reared. Their results show that larvae fed with an artificial diet have significantly more haemocytes circulating in their haemolymph and die considerably later when infested with viral pathogens compared to larvae fed with a plant-based diet. The artificial diet only marginally interacts with the density-dependent immune response. This shows that an artificial diet is suitable for investigating ecoimmunological questions related to DDP and other immune parameters.

The different studies in this Research Topic show that we are only beginning to understand the interaction between diet and health in insects and that a lot of knowledge gaps still need to be filled. In a changing environment, it is crucial to ensure that we understand 1) the different pressures experienced by insect species and 2) to what degree the insects can resist or tolerate them. A lack of diversity due to malnutrition or undernourishment will have a cascading effect within the trophic networks, thereby directly affecting biodiversity and food security. In light of the need for an integrated conservation approach when it comes to key pollinator species (4, 5), but also keeping in mind the important roles insects play within the ecosystem, the comprehension of what it means to “be a healthy insect” is an essential prerequisite to mitigate the challenges they experience, ultimately maintaining biodiversity and a healthier environment.

## Author contributions

All authors listed have made a substantial, direct, and intellectual contribution to the work and approved it for publication.
